# Sociodemographic and Geographic Variation in Awareness of Stroke Signs and Symptoms Among Adults — United States, 2017

**DOI:** 10.15585/mmwr.mm6944a1

**Published:** 2020-11-06

**Authors:** Sandra L. Jackson, Brian Legvold, Anjel Vahratian, Debra L. Blackwell, Jing Fang, Cathleen Gillespie, Donald Hayes, Fleetwood Loustalot

**Affiliations:** ^1^Division for Heart Disease and Stroke Prevention, National Center for Chronic Disease Prevention and Health Promotion, CDC; ^2^Rollins School of Public Health, Department of Epidemiology, Emory University, Atlanta, Georgia; ^3^Division of Health Interview Statistics, National Center for Health Statistics, CDC.

Stroke is the fifth leading cause of death in the United States (*1*). In 2017, on average, a stroke-related death occurred every 3 minutes and 35 seconds in the United States, and stroke is a leading cause of long-term disability (*1*). To prevent mortality or long-term disability, strokes require rapid recognition and early medical intervention (*2*,*3*). Common stroke signs and symptoms include sudden numbness or weakness of the face, arm, or leg, especially on one side; sudden confusion or trouble speaking; sudden trouble seeing in one or both eyes; sudden trouble walking, dizziness, or loss of balance; and a sudden severe headache with no known cause. Recommended action at the first sign of a suspected stroke is to quickly request emergency services (i.e., calling 9-1-1) (*2*). Public education campaigns have emphasized recognizing stroke signs and symptoms and the importance of calling 9-1-1, and stroke knowledge increased 14.7 percentage points from 2009 to 2014 (*4*). However, disparities in stroke awareness have been reported (*4*,*5*). Knowledge of the five signs and symptoms of stroke and the immediate need to call emergency medical services (9-1-1), collectively referred to as “recommended stroke knowledge,” was assessed among 26,076 adults aged ≥20 years as part of the 2017 National Health Interview Survey (NHIS). The prevalence of recommended stroke knowledge among U.S. adults was 67.5%. Stroke knowledge differed significantly by race and Hispanic origin (p<0.001). The prevalence of recommended stroke knowledge was highest among non-Hispanic White adults (71.3%), followed by non-Hispanic Black adults (64.0%) and Hispanic adults (57.8%). Stroke knowledge also differed significantly by sex, age, education, and urbanicity. After multivariable adjustment, these differences remained significant. Increasing awareness of the signs and symptoms of stroke continues to be a national priority. Estimates from this report can inform public health strategies for increasing awareness of stroke signs and symptoms.

NHIS is an annual survey of the civilian noninstitutionalized U.S. population. In 2017, NHIS included supplemental content in the sample adult interview that provided a list of five signs and symptoms and asked respondents to identify whether each was a symptom “that someone may be having a stroke.” Respondents also were asked to choose “the best thing to do right away” if “you thought someone was having a stroke.” One choice was to call 9-1-1.*

The prevalence of knowing each of the five signs and symptoms, to call 9-1-1 for a suspected stroke, and the combination of recommended stroke knowledge was estimated overall and by subgroup. Point estimates and corresponding variances were calculated using SAS-callable SUDAAN (version 11.0; RTI International), accounting for the complex sample design, and weighted to be nationally representative. Satterthwaite-adjusted chi-squared tests were used to assess significant (p<0.05) bivariate associations. Logistic regression models (including age, sex, race/ethnicity, education, county urbanicity [large metropolitan area, medium or small metropolitan area, and rural], and region [Northeast, Midwest, South, and West]) were used to generate adjusted prevalence ratios and 95% confidence intervals.

A majority of U.S. adults identified each of the individual signs and symptoms of stroke (Table 1). Prevalence was highest for “numbness of face, arm, leg, or side” (94.4%), “confusion or trouble speaking” (93.6%), and “trouble walking” (90.8%). “Sudden trouble seeing” was identified by 83.5%, and “sudden severe headache” by 76.5%. Awareness of calling 9-1-1 was high (96.3%). Prevalence of recommended stroke knowledge was 67.5%.

**TABLE 1:** Percentages (and standard errors)* of adults aged ≥20 years who knew stroke signs and symptoms and appropriate action to take in the event of a stroke, by sociodemographic and geographic characteristics — National Health Interview Survey, United States, 2017

Characteristic	% (Standard error)							
	Face, arm, leg, side numbness	Confusion, trouble speaking	Sudden trouble seeing	Trouble walking	Sudden severe headache	Knows all five stroke signs and symptoms	Knows to call 9-1-1	Knows all five signs and symptoms and to call 9-1-1^§^
**Total**	**94.4 (0.22)**	**93.6 (0.25)**	**83.5 (0.34)**	**90.8 (0.26)**	**76.5 (0.37)**	**69.1 (0.42)**	**96.3 (0.16)**	**67.5 (0.43)**
**Sex**								
Men	93.7 (0.32)	93.0 (0.36)	83.0 (0.48)	90.1 (0.38)	74.3 (0.53)	67.0 (0.60)	96.0 (0.23)	65.3 (0.60)
Women	95.0 (0.25)	94.2 (0.29)	83.9 (0.42)	91.4 (0.33)	78.6 (0.46)	71.2 (0.52)	96.6 (0.21)	69.6 (0.53)
p-value^†^	<0.001	0.005	0.123	0.006	<0.001	<0.001	0.039	<0.001
**Age group (yrs)**								
20–44	94.4 (0.32)	93.3 (0.39)	84.1 (0.53)	90.3 (0.40)	74.4 (0.57)	67.2 (0.63)	96.9 (0.24)	65.9 (0.63)
45–64	94.8 (0.35)	94.4 (0.35)	84.9 (0.47)	91.6 (0.40)	78.4 (0.57)	71.3 (0.64)	96.5 (0.25)	69.8 (0.65)
≥65	93.6 (0.37)	93.0 (0.38)	80.0 (0.62)	90.3 (0.45)	77.9 (0.66)	69.6 (0.71)	94.9 (0.33)	67.3 (0.74)
p-value	0.087	0.01	<0.001	0.02	<0.001	<0.001	<0.001	<0.001
**Race and Hispanic origin**								
White, non-Hispanic	96.6 (0.20)	96.5 (0.22)	86.8 (0.34)	93.5 (0.27)	79.0 (0.42)	73.0 (0.47)	96.7 (0.19)	71.3 (0.49)
Black, non-Hispanic	93.0 (0.74)	91.7 (0.90)	81.2 (1.12)	88.6 (0.84)	74.4 (1.18)	65.0 (1.33)	97.1 (0.40)	64.0 (1.34)
Other, non-Hispanic	91.1 (0.79)	88.6 (0.94)	78.5 (1.25)	87.8 (0.94)	71.6 (1.27)	63.5 (1.47)	94.7 (0.71)	61.9 (1.45)
Hispanic	88.0 (0.70)	86.0 (0.80)	74.2 (1.06)	82.7 (0.87)	70.7 (1.12)	59.6 (1.23)	95.2 (0.46)	57.8 (1.25)
p-value	<0.001	<0.001	<0.001	<0.001	<0.001	<0.001	<0.001	<0.001
**Level of education^¶^**								
Less than HS	85.3 (0.94)	82.7 (1.17)	70.3 (1.31)	80.2 (1.06)	67.9 (1.18)	56.7 (1.33)	93.5 (0.59)	54.8 (1.36)
HS or GED	92.9 (0.47)	92.4 (0.45)	79.8 (0.65)	88.7 (0.54)	74.4 (0.71)	65.1 (0.79)	96.0 (0.32)	63.4 (0.81)
Some college	96.3 (0.29)	95.7 (0.36)	85.9 (0.52)	93.2 (0.40)	78.1 (0.62)	71.1 (0.67)	96.9 (0.25)	69.4 (0.68)
College graduate	96.7 (0.23)	96.4 (0.25)	88.4 (0.43)	93.8 (0.34)	79.8 (0.54)	74.5 (0.62)	97.1 (0.22)	73.1 (0.61)
p-value	<0.001	<0.001	<0.001	<0.001	<0.001	<0.001	<0.001	<0.001
**County urbanicity**								
Large metropolitan counties	93.7 (0.27)	92.7 (0.34)	82.7 (0.43)	89.8 (0.34)	75.2 (0.49)	67.8 (0.54)	96.2 (0.22)	66.1 (0.54)
Medium or small metropolitan counties	95.6 (0.32)	95.2 (0.35)	85.1 (0.59)	92.4 (0.41)	79.2 (0.62)	71.4 (0.78)	96.7 (0.28)	69.9 (0.80)
Rural counties	94.8 (0.84)	94.1 (0.84)	83.2 (1.10)	91.3 (0.93)	76.4 (1.24)	70.2 (1.31)	96.0 (0.47)	68.3 (1.38)
p-value	0.004	<0.001	0.014	<0.001	<0.001	0.002	0.23	0.001
**Region**								
Northeast	94.9 (0.45)	93.4 (0.55)	83.9 (0.70)	90.2 (0.63)	77.6 (0.91)	70.4 (0.90)	96.3 (0.40)	69.0 (0.90)
Midwest	95.9 (0.33)	95.8 (0.31)	84.9 (0.63)	92.6 (0.48)	75.2 (0.67)	68.5 (0.79)	97.0 (0.30)	67.1 (0.84)
South	93.5 (0.43)	92.7 (0.53)	83.2 (0.60)	90.4 (0.46)	77.3 (0.65)	69.7 (0.75)	96.2 (0.27)	68.0 (0.77)
West	93.9 (0.41)	93.3 (0.45)	82.3 (0.73)	90.1 (0.53)	75.8 (0.80)	67.8 (0.93)	96.0 (0.36)	66.0 (0.92)
p-value	<0.001	<0.001	0.057	0.001	0.088	<0.001	0.153	0.123
Unweighted sample size	26,076	26,076	26,076	26,076	26,076	26,074	26,076	26,073

**Abbreviations:** GED = general educational development; HS = high school.

* Weighted percentages. “Don't know” responses are treated as “no”; “not ascertained and refused” responses are converted to blanks and are not included in the numerators or denominators.

^†^ P-values calculated from Satterthwaite-adjusted chi-squared tests.

^§^ The combination of knowing all five signs and symptoms of stroke and to call 9-1-1 is referred to as “recommended stroke knowledge.”

^¶^ Education was missing for 91 adults in the sample; these participants were omitted when education was assessed.

Awareness of individual signs and symptoms of stroke and recommended stroke knowledge differed significantly among subgroups (Table 1). The percentage of adults with recommended stroke knowledge ranged from 57.8% among Hispanic adults to 71.3% among non-Hispanic White adults and from 54.8% among adults with less than a high school education to 73.1% among college graduates. After multivariable adjustment, disparities in recommended stroke knowledge persisted by race and Hispanic origin and by education status. Smaller differences in the prevalence of recommended stroke knowledge were noted by sex, age, urbanicity, and region (Table 2).

**TABLE 2:** Adjusted prevalence ratios (and 95% CI)* of knowledge of stroke signs and symptoms and appropriate action to take in the event of a stroke, among adults aged ≥20 years — National Health Interview Survey, United States, 2017

Characteristic	Prevalence ratio (95% CI)							
	Face, arm, leg, side numbness	Confusion, trouble speaking	Sudden trouble seeing	Trouble walking	Sudden severe headache	Knows all five stroke signs and symptoms	Knows to call 9-1-1	Knows all five signs and symptoms and to call 9-1-1^†^
**Sex**								
Men versus women	0.99 (0.98–0.99)	0.99 (0.98–0.99)	0.99 (0.97–1.00)	0.99 (0.98–1.00)	0.95 (0.93–0.96)	0.94 (0.92–0.96)	0.99 (0.99–1.00)	0.94 (0.92–0.96)
**Age group (yrs)**								
20–44 versus ≥65	1.02 (1.00–1.03)	1.01 (1.00–1.02)	1.06 (1.04–1.08)	1.01 (0.99–1.02)	0.96 (0.94–0.98)	0.98 (0.96–1.01)	1.02 (1.01–1.03)	0.99 (0.97–1.02)
45–64 versus ≥65	1.02 (1.01–1.03)	1.02 (1.01–1.03)	1.07 (1.05–1.09)	1.02 (1.01–1.03)	1.01 (0.99–1.03)	1.03 (1.01–1.06)	1.02 (1.01–1.03)	1.04 (1.02–1.07)
**Race and Hispanic origin**								
Hispanic versus White, non-Hispanic	0.94 (0.93–0.95)	0.93 (0.91–0.94)	0.89 (0.87–0.92)	0.92 (0.91–0.94)	0.94 (0.91–0.97)	0.88 (0.84–0.91)	0.99 (0.98–1.00)	0.87 (0.83–0.91)
Black versus White, non-Hispanic	0.98 (0.96–0.99)	0.97 (0.95–0.98)	0.95 (0.92–0.97)	0.96 (0.94–0.98)	0.96 (0.92–0.99)	0.91 (0.88–0.95)	1.01 (1.00–1.02)	0.92 (0.88–0.96)
Other versus White, non-Hispanic	0.94 (0.92–0.96)	0.92 (0.90–0.94)	0.90 (0.87–0.93)	0.94 (0.92–0.96)	0.91 (0.88–0.95)	0.87 (0.83–0.91)	0.98 (0.96–0.99)	0.87 (0.83–0.91)
**Level of education^§^**								
Less than HS versus college degree	0.92 (0.90–0.93)	0.90 (0.88–0.92)	0.84 (0.81–0.87)	0.89 (0.87–0.91)	0.86 (0.83–0.89)	0.79 (0.75–0.83)	0.97 (0.96–0.98)	0.78 (0.74–0.82)
HS or GED versus college degree	0.96 (0.95–0.97)	0.96 (0.95–0.97)	0.91 (0.89–0.93)	0.95 (0.94–0.96)	0.93 (0.91–0.95)	0.88 (0.85–0.90)	0.99 (0.98–1.00)	0.87 (0.84–0.90)
Some college versus college degree	1.00 (0.99–1.00)	0.99 (0.99–1.00)	0.97 (0.96–0.99)	1.00 (0.98–1.01)	0.98 (0.96–1.00)	0.96 (0.93–0.98)	1.00 (0.99–1.00)	0.95 (0.93–0.97)
**County urbanicity**								
Rural versus large metropolitan	1.01 (0.99–1.03)	1.01 (0.99–1.03)	1.01 (0.98–1.03)	1.01 (0.99–1.03)	1.02 (0.99–1.05)	1.04 (1.00–1.08)	1.00 (0.99–1.01)	1.04 (1.00–1.08)
Medium or small metropolitan versus large metropolitan	1.02 (1.01–1.03)	1.02 (1.01–1.03)	1.02 (1.01–1.04)	1.02 (1.01–1.03)	1.05 (1.03–1.07)	1.05 (1.02–1.08)	1.01 (1.00–1.01)	1.05 (1.02–1.08)
**Region**								
Northeast versus Midwest	1.00 (0.99–1.01)	0.99 (0.97–1.00)	1.00 (0.98–1.03)	0.98 (0.97–1.00)	1.04 (1.01–1.07)	1.05 (1.01–1.09)	1.00 (0.99–1.01)	1.05 (1.01–1.09)
South versus Midwest	0.99 (0.98–1.00)	0.98 (0.97–1.00)	1.01 (0.99–1.03)	0.99 (0.98–1.01)	1.05 (1.02–1.07)	1.05 (1.02–1.09)	0.99 (0.99–1.00)	1.05 (1.02–1.08)
West versus Midwest	1.00 (0.99–1.01)	1.00 (0.99–1.01)	1.01 (0.98–1.03)	1.00 (0.98–1.01)	1.04 (1.01–1.07)	1.04 (1.00–1.08)	0.99 (0.99–1.00)	1.03 (1.00–1.07)
Unweighted sample size	25,985	25,985	25,985	25,985	25,985	25,983	25,985	25,982

**Abbreviations:** CI = confidence interval; GED = general educational development; HS = high school.

* Models included sex, age, race and Hispanic origin, education, county urbanicity, and region. “Don't know” responses on knowing the signs and symptoms of stroke were treated as no; all not ascertained and refused responses were treated as missing and excluded from these analyses.

^†^ The combination of knowing all five signs and symptoms of stroke and to call 9-1-1 is referred to as “recommended stroke knowledge.”

^§^ Education was missing for 91 adults in the sample; these participants were omitted when education was assessed.

## Discussion

Increasing awareness of signs and symptoms of stroke and the need to call 9-1-1 is vital to enable patients to quickly initiate stroke care and benefit from advances in treatment and systems of care (*6*,*7*). Although knowledge of most signs and symptoms of stroke, and for calling 9-1-1, were high, gaps in knowledge remain. Knowledge varied across geographic and sociodemographic subgroups. Consistent with overall prevalence reported for 2014 (66.2%) (*4*), approximately two thirds (67.5%) of U.S. adult respondents could identify the combination of recommended stroke knowledge in 2017.

Delays in recognizing stroke signs and symptoms might slow initiation of care. The symptom “sudden severe headache” had the lowest prevalence of awareness. This might be an artifact of its position in the survey questionnaire (the last listed symptom), or because headache is a symptom common to many conditions. Current stroke symptom awareness campaigns might inconsistently emphasize headache; some educational campaigns use incomplete acronyms, such as BE-FAST (balance, eyes, face, arms, speech, time), which does not reference headache.

The Healthy People 2020 goal for awareness of common stroke signs and symptoms (HDS-17) is 59.3% and for calling 9-1-1 is 94.7% (age-adjusted, using NHIS) (*8*). The weighted, but not age-adjusted, prevalence estimates reported here indicate that nationally and regionally, these targets might have been exceeded. However, consistent with previous work, this report demonstrates that awareness varies among some demographic groups (*4*,*5*). For example, multivariable results indicated that awareness of stroke signs and symptoms decreased with decreasing education. In addition, awareness was less prevalent among other race and Hispanic origin groups than among non-Hispanic White adults.

Previous studies have shown that stroke morbidity and mortality vary across populations and communities and disproportionately affect racial and ethnic minorities, persons with less education, and persons living in the Southeast (i.e., the “stroke belt”) (*1*). Among some subgroups, stroke mortality might be increasing, and overall, declines in stroke death rates have stalled in most states (*9*). The extent to which an increase in stroke knowledge could affect existing disparities and trends in stroke mortality is unknown.

Improvements in stroke outcomes depend on early recognition and timely initiation of care, as well as medical advances and care coordination. CDC’s Paul Coverdell National Acute Stroke Program^†^ aims to improve the continuum of care, including emergency services activation. In addition, the U.S. Department of Health and Human Services’ Million Hearts^§^ initiative aims to prevent 1 million heart attacks and strokes by 2022 through targeted community and health system interventions. The Get With The Guidelines-Stroke^¶^ program of the American Heart Association and the American Stroke Association has supported improvements in care, including evidence-based interventions such as tissue plasminogen activator (tPA) (*10*). Rapid recognition of stroke signs and symptoms and then immediately calling 9-1-1 increases the potential for ischemic stroke patients to quickly receive tPA, maximizing the health benefit.

The findings in this report are subject to at least five limitations. First, all data were self-reported and subject to recall and social desirability biases. Second, questions did not capture all potential stroke signs and symptoms. Third, close-ended (yes/no) questions might overestimate awareness. Fourth, no established standard is available for determining stroke awareness or how knowledge translates into appropriate action in response to a stroke, overall or across subgroups. Finally, the sample size was large, enabling detection of slight statistical differences, but no clear threshold exists for classifying meaningful differences in stroke knowledge to prompt earlier recognition and more timely care.

Primary prevention is central to promoting cardiovascular health and includes assessment and management of stroke risk factors (*7*). When strokes do occur, recognition of signs and symptoms and then calling 9–1-1 are needed to initiate care quickly to improve outcomes. This report identified overall high awareness of individual signs and symptoms, yet observed lower awareness for certain symptoms. Only approximately two thirds of adults surveyed had the combination of recommended stroke knowledge, and geographic variation and sociodemographic disparities remain. Focused public health efforts, community engagement, innovative strategies to tailor messaging, and continued advances in clinical care and coordination might help address stalled declines in stroke mortality (*9*). Increasing awareness of the signs and symptoms of stroke continues to be a national priority (*6*), and estimates from this report might be used to inform communication strategies.

**Summary**

**What is known about this topic?**

Awareness of stroke signs and symptoms and the need to call 9-1-1 when those occur can improve stroke outcomes.

**What is added by this report?**

During 2017, high levels of awareness of individual signs and symptoms of stroke and the need to call 9-1-1 when those occur were reported. However, only two thirds of U.S. adults had the combination of all recommended stroke knowledge, with sociodemographic and geographic variation.

**What are the implications for public health practice?**

Increasing awareness of the signs and symptoms of stroke continues to be a national priority. Estimates from this report might be used to inform communication strategies that improve awareness and reduce disparities.

## Footnotes

* The choices included “advise them to drive to the hospital,” “advise them to call their physician,” “call 9-1-1 (or another emergency number),” “call spouse or family member,” and “other.”

† https://www.cdc.gov/dhdsp/programs/stroke_registry.htm.

§ https://millionhearts.hhs.gov/.

¶ https://www.heart.org/en/professional/quality-improvement/get-with-the-guidelines/get-with-the-guidelines-stroke.
